# Q-fuzzy structure on JU-algebra

**DOI:** 10.12688/f1000research.160333.1

**Published:** 2025-01-20

**Authors:** Selamawit Hunie Gelaw, Birhanu Assaye Alaba, Mihret Alamneh Taye

**Affiliations:** 1 Bahir Dar University Department of Mathematics, Bahir Dar, Amhara, 79, Ethiopia

**Keywords:** JU-algebra, Q-Fuzzy JU-subalgebra, Q-Fuzzy JU- Ideal, Doubt Q-Fuzzy JU-algebra, Normal Q-Fuzzy JU-algebra, Level subsets

## Abstract

**Background:**

JU-algebras, an important class in abstract algebra, are extended here by incorporating fuzzy set theory to handle uncertainty in algebraic structures. In this study, we apply the concept of Q-fuzzy sets to JU-subalgebras and JU-ideals in JU-algebra.

**Method:**

The study defines Q-fuzzy JU-subalgebras and Q-fuzzy JU-ideals as subsets of a JU-algebra. It also explores lower and upper level subsets of these fuzzy structures to analyze their properties. Additionally, the concepts of Doubt and Normal Q-fuzzy JU-subalgebras and Q-fuzzy JU-ideals are introduced, offering a way to deal with varying degrees of uncertainty and regularity in these algebraic structures. Supportive concepts relevant to this study are presented, along with illustrative examples.

**Conclusion:**

The study introduces and defines new types of fuzzy structures in JU-algebras, such as Q-fuzzy JU-subalgebras and Q-fuzzy JU-ideals, enhancing classical JU-algebra theory. It also examines key properties of these structures, including their lower and upper level subsets, and investigates specific cases like Doubt and Normal Q-fuzzy structures, paving the way for further exploration of fuzzy algebra in mathematical and applied contexts.

## Introduction

After fuzzy subsets were defined by Ref. 1, several researchers explored Q-fuzzy sets in many algebraic structures. In 2001, Ref. 2 introduced the concept of Q-fuzzy subalgebras within BCI/BCK-algebras. Subsequently, Ref. 3 explored Q-fuzzy sets and their level subsets in UP-algebras. Additionally, Ref. 4 presented the concept of Doubt fuzzy ideals in BF-algebras, while Ref. 5 discussed Doubt Fuzzy Ideals of B-Algebras. In 2018, Ref. 6 further contributed by introducing the normalization of fuzzy B-ideals in B-algebra. The concept of JU-algebras was first proposed by Ref. 7 in 2020, and in 2022, Ref. 8 investigated ideals and subalgebras of JU-algebras.

Fuzzy sets in general can be applied in the area of medicine, computer science, cyber security and cryptography and others. In medical diagnosis, symptoms often lack clear-cut boundaries, making it challenging to classify them strictly. For example, blood sugar levels in diabetes can vary over a range, and fuzzy sets help model these varying degrees of health conditions. Using fuzzy set membership functions, a blood glucose level of 160 mg/dL could be assigned a degree of membership, such as 0.2 for low levels, 0.5 for medium levels, and 1 for high levels, based on predefined thresholds.

Consider a Q-fuzzy JU-algebra as follows:

Diabetes medical condition={Low(below140mg/dl,0.2),Medium(around160mg/dl,0.5),high(over200mg/dl,1)}

We can see that membership degree 0.2 mean that the patient medical condition is pre-diabetes, while membership degree 1 means the medical condition of an individual is hypoglycemia. Which helps the doctors to precisely measure and make robust decisions in diagnosis.

Despite its various applications, JU-algebra still requires further development. This motivates us to introduce the notion of a Q-fuzzy JU-subalgebras and a Q-fuzzy JU-ideals of JU-algebra and proved some results. We proved that the lower and upper level subsets of a Q-fuzzy JU-subalgebra or Q-fuzzy JU-ideal are also a JU-subalgebra or JU-ideals respectively. Additionally, we studied Doubt of Q-fuzzy JU-subalgebras and Doubt of Q-fuzzy JU-ideals of JU-algebra and investigated some of their properties. We proved that the Q-fuzzy subset of a JU-algebra is a Q-fuzzy JU-subalgebra or Q-fuzzy JU-ideal if and only if its complement is a Doubt Q-fuzzy subalgebra and Doubt of Q-fuzzy ideal, respectively. Furthermore, we proved that a normal Q-fuzzy JU-subalgebras or Q-fuzzy JU-ideals generated by any other Q-fuzzy JU-subalgebra or Q-fuzzy JU-ideals is also normal. We also studied the connection between Normal and Doubt of Q-fuzzy JU-subalgebras and Q-fuzzy JU-ideals of JU-algebra and obtained some important properties.

## Preliminaries

Definition 1 ^7^ *JU-algebra is an algebra* (X,◦,1) *of type* (2,0) *with a binary operation* ◦ *and a fixed element* 1 *if it holds* *,* ∀x,y,z∈X *:*

(a)
((x◦y)◦[(y◦z)◦(x◦z)]

(b)
1◦x=x

(c)
x◦y=1 *and* y◦x=1⇒x=y,∀x,y∈X

*In a JU-algebra X, we can define a partial ordering* “≤” *by Putting* x≤y *if and only* y◦x=1 .

Lemma 1 ^7, 9^ *If X is a JU-algebra, then the following hold for any* x,y,z∈X *:*

(a)
x≤y *implies* y◦z≤x◦z

(b)
x≤y *and* y≤x⇒x=y

(c)
x◦x=1

(d)
z◦(y◦x)=y◦(z◦x)

(e)
(x◦[(x◦y)◦y])=1

Definition 2 ^8^ *A nonempty subset S of a JU-algebra X is called a subalgebra of X if* x◦y∈S,∀x,y∈S.

Definition 3 ^8^ *A nonempty subset J of a JU-algebra X is said to be a JU-ideal of X if it satisfies:*

(a)
1∈J

(b)
x,x◦y∈J⇒y∈J,∀x,y∈X .

Definition 4 ^3^ *A Q-fuzzy set in a nonempty set X (or a Q-fuzzy subset of X) is an arbitrary function* η:X×Q→[0,1], *where Q is a nonempty set and [0, 1] is the unit segment of the real line.*

Definition 5 ^3^ *let η be a Q-fuzzy set in X,* ∀t∈ [0,1] *.The set* U(η;t,q)={x∈X|η(x)≥t} *is called an upper-level subset of η. And the set* L(η;t,q)={x∈X|η(x)≤t} *is called a lower-level subset of η.*

Definition 6 ^10^ *If η be a Q-fuzzy set in a set X. Then the complement denoted by* ηc *, is the Q-fuzzy subset of X given by* ηc(x,q)=1−η(x,q),∀x∈X *and* q∈Q.

Definition 7 ^4^ *let* µ *be a fuzzy subset of a JU-algebra X and* α∈[0,1]. *The fuzzy sets* ${µ}^{\alpha }$ *and* ${µ}_{\alpha }$ *of X are defined as follows:*

${µ}^{\alpha }=\min\left\{µ\left(x\right),\alpha \right\}$ *and* ${µ}_{\alpha }=\max\left\{µ\left(x\right),1-\alpha \right\},\forall x\in X.$ *It is evident that* ${µ}^{1}=\mu$ , $\mu^{0}=0$ *, and* $\mu_{1}=\mu ,\;\mu_{0}=\mu \;$

Definition 8 ^6^ *A fuzzy set μ of a JU-algebra X is said to be normal if there exists* x∈X *such that* µ(x)=1 .

## Q-Fuzzy subalgebras of a JU-algebra

In this section we discuss and investigate a new notion of Q-fuzzy JU-subalgebras in JU-algebras and study their related properties.

Definition 9 *Let X be a JU-algebra, a Q-fuzzy set η in X is called Q-fuzzy JU-subalgebra of X if for any* x,y∈X

η(x◦y,q)≥min{η(x,q),η(y,q)},∀q∈Q.

Example 1 *Let* X={1,2,3,4} *in which* ◦ *is defined by the following table (* *Table 1* *)*. ^7^ *Clearly,* (X,◦,1) *is a JU-algebra. Let* $Q=\left\{q_{1},q_{2},q_{3}\right\}$ *and we define a Q-fuzzy set* η:Q×X→[0,1] : *by Routine calculation gives that* η *is a Q-fuzzy JU-subalgebra of X (* *Table 2* *).*

**Table 1.** 

◦	**1**	**2**	**3**	** 4**
**1**	1	2	3	4
**2**	1	1	4	1
**3**	1	1	1	1
**4**	1	4	4	1

**Table 2.** 

◦	***q*_1_**	***q*_2_**	***q*_3_**
**1**	0.9	0.7	0.8
**2**	0.6	0.5	0.6
**3**	0.4	0.3	0.5
**4**	0.5	0.4	0.7

Lemma 2 *If η is a Q-fuzzy JU-subalgebra of a JU-algebra X. Then* η(1,q)≥η(x,q),∀x∈X *and* ∀q∈Q .

Proof:
*Suppose η be a Q-fuzzy JU-subalgebra of a JU-algebra X.*

*Since,* η(1,q)=η(x◦x,q) ............... *(Lemma no. 1 c)*

     

≥min{η(x,q),η(x,q)}

     

=η(x,q)

Definition 10 *Let η be a Q-fuzzy set in a JU-algebra X,* ∀t∈[0,1]. *The set* η={x∈X|η(x,q)≥t} *is called an upper-level subset of η. And the set* $\eta_{t}=\left\{x\in X|\eta \left(x\right)\leq t\right\}$ *is called a lower-level subset of η.*

*Clearly _,_* ηt∪ηt=X *for any* t∈[0,1] *if* $t_{1}\leq t_{2}$ *, then* $\eta_{t_{1}}\subseteq \eta_{t_{2}}$ *but if* $t_{2}\leq t_{1},$ *then* $\eta_{t_{2}}\subseteq \eta_{t_{1}}$ _._

Theorem 3 *A Q-fuzzy set η of a JU-algebra X is a Q-fuzzy subalgebra if and only if for every* t∈[0,1],q∈Q *, and* ηt *is a JU-subalgebra of X.*

**Proof:** *Suppose that* η *is a Q-fuzzy subalgebra of a JU-algebra X and* $\eta_{t}\neq \emptyset$ *. For any* $x,y\in \eta_{t}$ *such that* η(x,q)≤t *and* η(y,q)≤t *implies that*

$$\begin{array}{l} \eta \left(x◦y,q\right)\leq \min\left\{\eta \left(x,q\right),\eta \left(y,q\right)\right\}\\ \;\leq \min\left\{t,t\right\}=t\\ \;\Rightarrow x◦y\in \eta_{t} \end{array}$$

*Hence,* $\eta_{t}$ *is a subalgebra of X.*

*Conversely, if η _t_ is a subalgebra of X. Let x*, *y* ∈ *η _t_ and there exist* $t_{1},t_{2}\in \left[0,1\right]$ *such that* $\eta \left(x,q\right)\leq t_{1}$ *and* $\eta \left(y,q\right)\leq t_{2}$ .

*Take,* $=\min\left\{t_{1},t_{2}\right\}$ *. By assumption* $\eta_{t}$ *is a subalgebra of X, we have* $\;x◦y\in \eta_{t}$ *then* η(x◦y)≤t=min{η(x,q),η(y,q)}η *is a Q-fuzzy JU-subalgebra of X.*

Theorem 4 *A Q-fuzzy set η of a JU-algebra X is a Q-fuzzy subalgebra if and only if for every* t∈[0,1] *and* q∈Q *,* ηt *is a JU-subalgebra of X.*

Proof:
*It is straight forward by theorem 3.*

Definition 11 *A Q-fuzzy set η of a JU-algebra X is called a Doubt Q-fuzzy JU-subalgebra of X if* ∀x,y∈X *and* q∈Q

η(x+y,q)≤max{η(xq)η(y,q)}

Example 2 *Let* X={1,2,3,4} *in which* ◦ *is defined by the following table (* *Table 3* *).* ^7^

**Table 3.** 

◦	**1**	**2**	**3**	** 4**
**1**	1	2	3	4
**2**	2	1	2	2
**3**	1	2	1	3
**4**	1	2	1	1

*Clearly,* (X,◦,1) *is a JU-algebra. Let* $Q=\left\{q_{1},q_{2}\right\}$ *and we define a Q-fuzzy set* η:Q×X→[0,1] *by:*

*Routine calculation gives that η is a Doubt Q-fuzzy JU-subalgebra of X (* *Table 4* *).*

**Table 4.** 

◦	***q*_1_**	***q*_2_**
**1**	0.3	0.1
**2**	0.5	0.6
**3**	0.8	0.7
**4**	0.9	0.8

Lemma 5 *If* η *is a Doubt Q-fuzzy JU-subalgebra of a JU-algebra X. Then* η(1,q)≤η(x,q),∀x∈X *and* ∀q∈Q .

Proof:
*Suppose η be a Doubt Q-fuzzy JU-subalgebra of a JU- algebra X.*

*Since,* (1,q)=η(x◦x,q) *.................. (Lemma no. 1 c)*

     

≥max{η(x,q),η(x,q)}

     

=η(x,q)

Theorem 6 *For every subalgebra of a JU-algebra X, it can be realized as a level subalgebra of some Doubt Q-fuzzy JU-subalgebra of X.*

Proof:
*Let X be a subalgebra of a JU-algebra X, and let η be a Q-fuzzy set in X defined by:*

$$\eta \left(x,q\right)=\left\{ \begin{array}{ll} t, & \text{if}\;x\in X\;\text{and}\;q\in Q\\ 0, & \text{if}\;x\notin X\;\text{and}\;q\in Q \end{array}\right.$$

*Where* t∈[0,1] *is fixed. Clearly,* ηt∪ηt=X .

*We went to prove that η is a Doubt Q-fuzzy subalgebra of X.*

*If* x,y∈X *and* q∈Q *then* x◦y∈X

$$\begin{array}{l} \eta \left(x,q\right)=\eta \left(x◦y,q\right)=t\\ \eta \left(x◦y,q\right)\leq \max\left\{\;\eta \left(x,q\right),\eta \left(y,q\right)\right\} \end{array}$$

If x,y∉X *and* q∈Q *then* η(x,q)=η(y,q)=0

η(x◦y,q)≤max{η(x,q),η(y,q)}=0

*If at most one of* x,y∈X *and* q∈Q *then at least one of* η(x,q) *and* η(y,q) *is equal to t. Thus*

$$\begin{array}{l} \max\left\{\eta \left(x,q\right),\eta \left(y,q\right)\right\}=t\\ \Rightarrow \left(x◦y,q\right)\leq t. \end{array}$$

Theorem 7 *A Q-fuzzy subset η of a JU-algebra X is a Q-fuzzy subalgebra of X if and only if its complement η ^c^ is a Doubt Q-fuzzy subalgebra of X.*

Proof:
*Let η be a Q-fuzzy subalgebra of X and let x*, *y* ∈ *X.*

*Then*

$$\begin{array}{l} \eta^{c}\left(x◦y,q\right)\leq 1-\min\left\{\eta \left(x,q\right),\eta \left(y,q\right)\right\}\\ \;=\max\left\{1-\eta \left(x,q\right),1-\eta \left(y,q\right)\right\}\\ \;=\max\;\eta^{c}\left(x,q\right),\eta^{c}\left(y,q\right) \end{array}$$

*Conversely, suppose is a Doubt Q-fuzzy subalgebra of X.*

$$\begin{array}{l} 1-\eta \left(x◦y,q\right)=\eta^{c}\left(x◦y,q\right)\leq \max\left\{\eta^{c}\left(x,q\right),\eta^{c}\left(y,q\right)\right\}\\ =\max\left\{1-\eta \left(x,q\right),1-\eta \left(y,q\right)\right\}\\ =1-\min\left\{\eta \left(x,q\right),\eta \left(y,q\right)\right\}\\ \Rightarrow \eta \left(x◦y,q\right)\geq \min\left\{\eta \left(x,q\right),\eta \left(y,q\right)\right\} \end{array}$$

Definition 12 *A Q-fuzzy JU-subalgebra η of X is said to be normal if there exists* x∈X *such that* η(x,q)=1

Example 3 *Consider* X={1,2,3,4,5}, *we construct the following table.*

*Clearly,* ( *X*, ◦, 1) *is a JU-algebra (* *Table 5* *)*. ^7^

**Table 5.** 

◦	**1**	**2**	**3**	**4**	** 5**
**1**	1	2	3	4	5
**2**	1	1	3	4	5
**3**	1	2	1	4	4
**4**	1	1	3	1	3
**5**	1	1	1	1	1

*Let* $Q=\left\{q_{1},q_{2}\right\},$ *given a Q-fuzzy set* η:Q×X→[0,1] *then it gives η is a normal Q-fuzzy JU-subalgebra of X(* *Table 6* *).*

**Table 6.** 

◦	***q*_1_**	***q*_2_**
**1**	1	1
**2**	0.8	0.9
**3**	0.7	0.6
**4**	0.5	0.4
**5**	0.3	0.1

Lemma 8 *If η is a normal Q-fuzzy JU-subalgebra of a JU-algebra X, then* η(1,q)=1

Proof:
*Suppose η be normal Q-fuzzy JU-subalgebra of a JU-algebra X.*

*Since,* η(1,q)≥η(x,q)=1 *.......................(* *Lemma 2* *)*

⇒η(1,q)≥1 B *ut,* η(1,q)≤1

*Therefore,* η(1,q)=1 .

Theorem 9 *For a Q-fuzzy JU-subalgebra of η of X, we can generate a normal fuzzy subalgebra of X which contains η.*

***Proof:** Let η be a Q-fuzzy JU-subalgebra of X. Define a Q-fuzzy subset* $\eta^{*}$ *of X as:*

$$\eta^{*}\left(x,q\right)=\eta \left(x,q\right)+\eta^{c}\left(1,q,\right),\forall_{x}\in X.$$

*Let* x,y∈X *,* $\eta^{*}\left(x◦y,q\right)=\eta \left(x◦y,q\right)+\eta^{c}\left(1,q\right)$

$$\begin{array}{l} \geq \min\left\{\eta \left(x,q\right),\eta \left(y,q\right)\right\}+\eta^{c}\left(1,q)\right)\\ =\min\left\{\eta \left(x,q\right)+\eta^{c}\left(1,q\right),\eta \left(y,q\right)+\eta^{c}\left(1,q\right)\right\}\\ =\min\left\{\eta^{*}\left(x,q\right),\eta^{*}\left(y,q\right)\right\} \end{array}$$

*And*

$$\begin{array}{l} \eta^{*}\left(1,q\right)=\eta \left(1,q\right)+\eta^{c}\left(1,q\right)\\ =\eta \left(1,q\right)+1-\eta \left(1,q\right)=1 \end{array}$$

⇒ *is normal Q-fuzzy JU-algebra of X. Clearly,* $\eta \subseteq \eta^{*}$ *thus* $\eta^{*}$ *is a normal JU-subalgebra of X which contains η.*

Corollary 10 *If η itself is normal then* $\eta =\eta^{*}$ *If η is a Q-fuzzy JU-subalgebra of X then* $\left(\eta^{*}\right)*=\eta^{*}$

Proof:
*It is straight forward by* *theorem 9*.

Theorem 11 *Let η be a Q-fuzzy JU-subalgebra of X. If η contains a normal Q-fuzzy JU-subalgebra of X generated by any other Q-fuzzy JU-subalgebra of X then η is normal.*

Proof:
*Let λ be a Q-fuzzy JU-subalgebra of X. by* *theorem 9* *and* $\lambda^{*}$ *is a normal Q-fuzzy JU-subalgebra of X. i.e.* $\lambda^{*}=1$ *by* *Lemma 8* *. Then let η is a Q-fuzzy JU-subalgebra of X such that* $\lambda^{*}\subseteq \eta$ *then*

$$\eta \left(x,q\right)\geq \lambda^{*}\left(x,q\right),\forall x\in X$$

*Put x* = 1 ⇒ *η* (1, *q*) *≥* $\lambda^{*}$ = 1

⇒η(1,q)≥1 *. Hence η is normal.*

Theorem 12 *Let η be a Q-fuzzy JU-subalgebra of X and let* f:[1,η(1,q)]→f[0,1] *be an increasing function. Define a Q-fuzzy set* η:X→[0,1] *by* η=f(η(x,q)),∀x∈X *then*

i.
$\eta^{f}$ *is a Q-fuzzy JU-subalgebra of X.*
ii. *If* f(η(1,q))=1 *then* $\eta^{f}$ *is normal*
iii. *If* f(t)≥t,∀t∈[1,η(1,q)] *then* $\eta \subseteq \eta^{f}$

Proof:

i.
$$\begin{array}{l} \eta^{f}\left(x◦y,q\right)=f\left(\eta \left(x◦y,q\;\right)\right)\\ \;\geq f\left(\min\left\{\eta \left(x,q\right),\eta \left(y,q\right)\right\}\right)\\ \;=\min\left\{f\left(\eta \left(x,q\right)\right),f\left(\eta \left(y,q\right)\right)\right\}\\ \;=\min\;\eta^{f}\left(x,q\right),\eta^{f}\left(y,q\right) \end{array}$$

$\Rightarrow \eta^{f}$ *is a Q-fuzzy subalgebra of X.*
ii. *If* f(η(1,q))=1
$$\Rightarrow \eta^{f}\left(1,q\right)=1$$

$\Rightarrow \eta^{f}$ *is normal*
iii. *Let* f(t)≥t,∀t∈[1,η(1,q)]

*Then*
$$\begin{array}{l} \Rightarrow \eta^{f}\left(x,q\right)=f\left(\eta \left(x,q\right)\right)\\ \geq \eta \left(x,q\right),\forall x\;f\in X \end{array}$$
*Hence* $\eta \subseteq \eta^{f}$ .

Theorem 13 *Let η be a Doubt Q-fuzzy JU-subalgebra of X and let* g:[1,η(1,q)]→[0,1] *be a decreasing function. Define a Q-fuzzy set* η:X→[0,1] *by* $\eta^{g}=g\left(\eta \left(x,q\right)\right),\forall x\in X$ *then*

i.
$\eta^{g}$ *is a Doubt Q-fuzzy JU-subalgebra of X.*
ii. *If* g(η(1,q))=1 *then* ηg *is normal*
iii. *If* g(t)≤t,∀t∈[1,η(1,q)] *then* $\eta^{g}\subseteq \eta$

Proof:

i. *Suppose η be a Doubt Q-fuzzy JU-subalgebra of X. Then*
$$\begin{array}{l} =\max\left\{g\left(\eta \left(x,q\right)\right),g\left(\eta \left(y,q\right)\right)\right\}\\ =\max\left\{\eta^{g}\left(x,q\right),\eta^{g}\left(y,q\right)\right\}\\ =\eta^{g}\left(x◦y,q\right)=\left(\eta \left(x◦y,q\right)\right)\\ \leq g\left(\max\left\{\;\eta \left(x,q\right),\eta \left(y,q\right)\right\}\right) \end{array}$$

$\Rightarrow \eta^{g}$ *is a Doubt Q-fuzzy subalgebra of X*.
ii. *If* g(η(1,q))=1
$$\Rightarrow \eta^{g}\left(1,q\right)=1$$

$\Rightarrow \eta^{g}$ *is normal*.
iii. *let* g(t)≤t,∀t∈[1,η(1,q)]

*Then* $\eta^{g}\left(x,q\right)=g\left(\eta \left(x,q\right)\right)$
≤η(x,q),∀x∈X
*Hence* $\eta^{g}\subseteq \eta$

## Q-Fuzzy Ideal of JU-algebras

In this section we discuss and investigate new notion of Q-fuzzy JU-ideals in JU-algebras and study their related properties.

Definition 13 *Let X be a JU-algebra, a Q-fuzzy set η in X is called Q-fuzzy JU-ideal of X if it satisfies the following conditions:*

(a)
η(1,q)≥η(x,q)

(b)
η(y,q)≥min{η(x,q),η(x◦y,q)},∀x,y∈Xand∀q∈Q .

Example 4 *(See table 3.1 from* *example 3* *) consider* X={1,2,3,4} *, and let* $Q=\left\{q_{1},q_{2}\right\},$ *given a Q-fuzzy set* η:Q×X→[0,1] *by Routine calculation gives that η is a Q-fuzzy JU-ideal of X (* *Table 7* *).*

Theorem 14 *Every Q-fuzzy JU-ideals of a JU-algebra X is order reversing.*

Proof:
*Let* η *be a Q-fuzzy JU-ideals of a JU-algebra X and for any* x,y∈X *be such that* x≤y *, then* y◦x=1

$$\begin{array}{l} \eta \left(x,q\right)\geq \min\left\{\eta \left(y◦x,q\right),\eta \left(y,q\right)\right\},\forall q\;\epsilon \;Q\\ \;=\min\left\{\eta \left(1,q\right),\eta \left(y,q\right)\right\}=\eta \left(y,q\right) \end{array}$$

Theorem 15 *If η is a Q-fuzzy JU-ideals of X, then η* (( *x ◦ y*) *◦ y*, *q*) *≥ η*( *x*, *q*).

Proof:
*Since* ( *x◦* [( *x◦y*) *◦y*]) = 1 *.......................... (Lemma no.1e)*

*Now,* η((x◦y)◦y,q)≥min{η(x,q),η(x◦[(x◦y)◦y])} , ∀q∈Q=min{η(x,q),η(1,q)}=η(x,q)

Theorem 16 *A Q-fuzzy ideal of a JU- algebra X is Q-fuzzy JU-subalgebra when* η(x◦(x◦y),q)≥η(y,q), *for any* x,y∈X *and* ∀q∈Q .

Proof:
*Suppose* η *is a Q-fuzzy ideal of a JU- algebra X and* η(x◦(x◦y),q)≥η(y,q). *Then for any* x,y∈X *and* ∀q∈Q .

$$\begin{array}{l} \eta \left(x◦y,q\right)\geq \min\left\{\eta \left(x,q\right),\eta \left(x◦\left(x◦y\right),q\right)\right\}\\ \;\geq \min\left\{\eta \left(x,q\right),\eta \left(y,q\right)\right\}. \end{array}$$

Theorem 17 *Let η be a Q-fuzzy set in a JU-algebra X and let* t∈[0,1]. *Then η is a Q-fuzzy JU-ideal of X if and only if the upper level subset* $\eta^{t}$ *is a JU-ideal of X.*

Proof:
*Suppose η be a Q-fuzzy JU-ideal in a JU-algebra X and* $\eta^{t}\neq \emptyset$

*Let* $x,x◦y\in \eta_{t}$ *and* ∀q∈Q *implies that* η(x,q)≥t *and* η(x◦y,q)≥t *then* η(y,q)≥min{η(x,q),η(x◦y,q)}≥min{t,t}=t

*This implies that y* $\in \eta^{t}$ *and hence* $\eta^{t}$ *is a JU-ideal of X.*

*Conversely, assume that the upper level subset η ^t^ is a JU-ideal of X.*

*Further, let we assume that there exist some x*_0_ ∈ *X such that* $\eta \left(1,q\right)<\eta \left(x_{0},q\right)$

*Take* $s=\frac{1}{2}\left[\eta \left(1,q\right)*\eta \left(x_{0},q\right)\right]$

$$\Rightarrow \eta \left(1,q\right)<s<\eta \left(x_{0},q\right)$$

$x_{0}\in \eta_{s}$ *and* $1\notin \eta_{s}$ *, which is contradict to our assumption that* $\eta_{s}$ *is a JU-ideal of X.*

*Therefore,* η(1,q)≥η(x,q),∀x∈X .

*And*

*assume that* $x_{0},y_{0}\in X$ *such that*

$$\eta \left(y_{0},q\right)<\min\left\{\eta \left(x_{0},q\right),\eta \left(x_{0}◦y_{0},q\right)\right\}$$

*Take* $s=\frac{1}{2}\left[\eta \left(y_{0},q\right)+\min\left\{\eta \left(x_{0},q\right),\eta \left(x_{0}◦y_{0},q\right)\right\}\right]$

$$\begin{array}{l} \Rightarrow \eta \left(y_{0},q\right)<s<\min\left\{\eta \left(x_{0},q\right),\eta \left(x_{0}◦y_{0},q\right)\right\}\\ \Rightarrow s>\eta \left(y_{0},q\right)\;\text{and}\;s<\min\left\{\eta \left(x_{0},q\right),\eta \left(x_{0}◦y_{0}\right),q\right\}\\ \Rightarrow s>\eta \left(y_{0},q\right),s<\eta \left(x_{0},q\right),\text{and}\;\;s<\eta \left(x_{0}◦y_{0},q\right) \end{array}$$

$y_{0}\notin \eta_{s}$ *, a contradiction, since* $\eta_{s}$ *is a JU-ideal of X. Therefore,* $\eta \left(y_{0},q\right)\geq \min\left\{\eta \left(x_{0},q\right),\eta \left(x_{0}◦y_{0}\right),q\right\}$ *for any* x,y∈X *and* ∀q∈Q.

Theorem 22 *For a Q-fuzzy JU-ideal of η of X, we can generate a normal fuzzy ideal of X which contains η.*

Proof:
*Let η be a Q-fuzzy JU-ideal of X. Define a Q-fuzzy subset* $\eta^{*}$ *of X as:*

$$\eta^{*}\left(x,q\right)=\eta \left(x,q\right)+\eta_{c}\left(1,q\right),\forall x\in X.$$

Let $x,y\in X,\eta *\left(1,q\right)=\eta \left(1,q\right)+\eta_{c}\left(1,q\right)$

$\geq \eta \left(x,q\right)+\eta_{c}\left(1,q\right)=\eta^{*}\left(x,q\right)$ *And*

$$\begin{array}{l} \eta^{*}\left(y,q\right)=\eta \left(y,q\right)+\eta_{c}\left(1,q\right)\\ \;\geq \min\left\{\eta \left(x,q\right),\eta \left(x◦y,q\right)\right\}+\eta_{c}\left(1,q\right)\\ \;=\min\left\{\eta \left(x,q\right)+\eta_{c}\left(1,q\right),\eta \left(x◦y,q\right)+\eta_{c}\left(1,q\right)\right\}\\ \;=\min\{\eta^{*}\left(x,q\right),\eta^{*}\left(x◦y,q\right)\}\;\text{Also }\;\eta^{*}\left(1,q\right)\\ \;=\eta \left(1,q\right)+\eta_{c}\left(1,q\right)\\ \;=\eta \left(1,q\right)+1-\eta \left(1,q\right)=1 \end{array}$$

$\Rightarrow \eta^{*}$ *is normal Q-fuzzy JU-algebra of X*.

*Clearly* $\eta \subseteq \eta^{*}$ *thus* $\eta^{*}$ *is a normal JU-ideal of X which contains η*.

Corollary 23 If *η itself is normal then* $\eta =\eta^{*}$ *. And if* η *is a Q-fuzzy JU-ideal of X then* $\left(\eta^{*}\right)*=\eta^{*}$

Proof:
*It is straight forward by* theorem 22.

Theorem 24 *Let η is a Q-fuzzy JU-ideal of X. If η contains a normal Q-fuzzy JU-ideal of X generated by any other Q-fuzzy JU- ideal of X then η is normal.*

Proof:
*Let λ be a Q-fuzzy JU- ideal of X. By* *theorem 22* λ∗ *is a normal Q-fuzzy JU-ideal of X. i.e.,* λ∗ = 1 *by Lemma 21*

*Let η is a Q-fuzzy JU-ideal of X such that* λ∗⊆η *then* $\eta \left(x,q\right)\geq \lambda^{*}\left(x,q\right),\forall x\in X$ *Put* x=1

$$\Rightarrow \eta \left(1,q\right)\geq \lambda^{*}=1$$

⇒η(1,q)≥1. *but*, η(1,q)≤1

Therefore, η(1,q)=1.

Theorem 25 *Let η be a Q-fuzzy JU- ideal of X and let* f:[1,η(1,q)]→[0,1] *be an increasing function. Define a Q-f fuzzy set* $\eta_{f}:X\to \left[0,1\right]$ *by* η=f(η(x,q)),∀x∈X *then*

i.
$\eta_{f}$ *is a Q-fuzzy JU- ideal of X.*
ii. *If* f(η(1,q))=1 *then* ηf *is normal*
iii. *If* f(t)≥t,∀t∈[1,η(1,q)] *then* η⊆ηf

Proof:

i.
$$\begin{array}{l} \eta_{f}\left(1,q\right)=f\left(\eta \left(1,q\right)\right)\\ \;\geq f\left(\eta \left(x,q\right)\right)\\ \;=\eta_{f}\left(x,q\right) \end{array}$$
*Also* $\eta_{f}\left(y,q\right)=f\left(\eta \left(y,q\right)\right)$
$$\begin{array}{l} \geq f\left(\min\left\{\eta \left(x,q\right),\eta \left(x◦y,q\right)\right\}\right)\\ =\min\left\{f\left(\eta \left(x,q\right)\right),f\left(\eta \left(x◦y,q\right)\right)\right\}\\ =\min\left\{\eta_{f}\left(x,q\right),\eta_{f}\left(x◦y,q\right)\right\} \end{array}$$

$\Rightarrow \eta_{f}$ *is a Q-fuzzy JU-ideal of X.*
ii. *If* f(η(1,q))=1
$$\Rightarrow \eta_{f}\left(1,q\right)=1$$

$\Rightarrow \eta_{f}$ *is normal*
iii. *Let* f(t)≥t,∀t∈[1,η(1,q)]

*Then* $\eta_{f}\left(x,q\right)=f\left(\eta \left(x,q\right)\right)$

≥η(x,q),∀x∈X

*Hence* $\eta \subseteq \eta_{f}$ .

Theorem 26 *Let η be a Doubt Q-fuzzy JU- ideal of X and let* g:[1,η(1,q)]g→[0,1] *be a decreasing function. Define* g *a Q-fuzzy set* η:X→[0,1]byη=g(η(x,q)),∀x∈X *then*

i.
$\eta^{g}$ *is a Doubt Q-fuzzy JU-ideal of X.*
ii. *If* g(η(1,q))=1 *then* $\eta^{g}$ *is normal*
iii.
g(t)≤t,∀t∈[1,η(1,q)] *then* $\eta^{g}\subseteq \eta$

Proof:

i. *Suppose η is a Doubt Q-fuzzy JU-ideal of X. Then*
$$\begin{array}{l} \eta^{g}\left(1,q\right)=g\left(\eta \left(1,q\right)\right)\\ \leq g\left(\eta \left(x,q\right)\right)=\eta^{g}\left(x,q\right) \end{array}$$
*Also*
$$\begin{array}{l} \eta^{g}\left(y,q\right)=g\left(\eta \left(y,q\right)\right)\\ \;\leq g\left(\max\left\{\eta \left(x,q\right),\eta \left(x◦y,q\right)\right\}\right)\\ \;=\max\left\{g\left(\eta \left(x,q\right)\right),g\left(\eta \left(x◦y,q\right)\right)\right\}\\ \;=\max\left\{\eta^{g}\left(x,q\right),\eta^{g}\left(x◦y,q\right)\right\} \end{array}$$

$\Rightarrow \eta^{g}$ *is a Doubt Q-fuzzy ideal of X.*
ii. *If* g(η(1,q))=1
$$\Rightarrow \eta^{g}\left(1,q\right)=1$$

$\Rightarrow \eta^{g}$ *is normal*
iii. *let* g(t)≤t,∀t∈[1,η(1,q)] t *hen*
$$\begin{array}{l} \eta^{g}\left(x,q\right)=g\left(\eta \left(x,q\right)\right)\\ \;\leq \eta \left(x,q\right),\forall x\in X \end{array}$$

$\text{Hence}\;\eta^{g}\subseteq \eta$

**Table 7.** 

◦	***q*_1_**	***q*_2_**
**1**	0.6	0.8
**2**	0.3	0.5
**3**	0.2	0.4
**4**	0.2	0.1

## Conclusions

In this study, we discussed the Q-fuzzy JU-subalgebras and Q-fuzzy JU-ideals of JU-algebra and investigated several related properties. We proved that the lower and upper level subsets of a Q-fuzzy JU-subalgebra or Q-fuzzy JU-ideal are also a JU-subalgebra or JU-ideals. Additionally, we studied Doubt of Q-fuzzy JU-subalgebras and Doubt of Q-fuzzy JU-ideals of JU-algebra and investigated some of their properties. We proved that the Q-fuzzy subset of a JU-algebra is a Q-fuzzy JU-subalgebra or Q-fuzzy JU-ideal if and only if its complement is a Doubt Q-fuzzy subalgebra and Doubt of Q-fuzzy ideal, respectively. Furthermore, we proved that a normal Q-fuzzy JU-subalgebras or Q-fuzzy JU-ideals generated by any other Q-fuzzy JU-subalgebra or Q-fuzzy JU-ideals is also normal. We also studied the connection between Normal and Doubt of Q-fuzzy JU-subalgebras and Q-fuzzy JU-ideals of JU-algebra and obtained some important properties. The findings of this research are expected to enrich the theory of Q-fuzzy JU-subalgebras and Q-fuzzy JU-ideals, based on Q-fuzzy sets, and will serve as a base for further study on this concept.

## Author contributions

Selamawit Hunie Gelaw: Conceptualization, data curation, formal analysis, Investigation, Methodology, and original draft preparation. Birhanu Assaye Alaba and Mihret Alamneh Taye: Conceptualization, Methodology, supervision, Validation, writing review and editing. All authors have read and agreed to the published version of the manuscript.

## Ethics approval and consent to participate

Not applicable.

## Consent for publication

Not applicable.

## Data Availability

No data is associated with this article.
