# Supplementary material for: Q-fuzzy structure on JU-algebra
Source: F1000Res. 2025 Jan 20;14:109. [Version 1] doi: 10.12688/f1000research.160333.1 (PMC11937783; doi:10.12688/f1000research.160333.1)
Supplement: Peer review history

# Reviewer report: Reviewer response for version 1

**DOI:** 10.5256/f1000research.176217.r368443

**Published:** 2025-03-25

**Authors:** Necati Olgun

**Affiliations:** 1 Gaziantep University, Gaziantep, Turkey

JU-algebras is an important class in abstract algebra.

 In the article JU-algebras have extended by incorporating fuzzy set theory to handle uncertainty in algebraic structures.  In this study, authors have applied the concept of Q-fuzzy sets to JU subalgebras and JU-ideals in JU-algebra and defined Q-fuzzy JU-subalgebras and Q-fuzzy JU-ideals as subsets of a JU-algebra also explored lower and upper level subsets of these fuzzy structures to analyze their properties.

 Additionally, the concepts of Doubt and Normal Q-fuzzy JU-subalgebras and Q-fuzzy JU ideals are introduced, offering a way to deal with varying degrees of uncertainty and regularity in these algebraic structures.

 ​​​​​The paper is original and has no errors from a mathematical point of view.

 However, it needs some minor corrections. For example, there are definitions at the beginning of the paper but no relevant examples. There is no information about the definition of fuzzy set. Again, there is no definition and result from references 1 and 2. The number of references in the article is very low. You can look at the studies done in recent years and add them to the reference section.

Is the work clearly and accurately presented and does it cite the current literature?

Yes

If applicable, is the statistical analysis and its interpretation appropriate?

Partly

Are all the source data underlying the results available to ensure full reproducibility?

Yes

Is the study design appropriate and is the work technically sound?

Yes

Are the conclusions drawn adequately supported by the results?

Yes

Are sufficient details of methods and analysis provided to allow replication by others?

Yes

Reviewer Expertise:

Abstract Algebra, Sets, Groups , Rings, Modules, Fuzzy sets

I confirm that I have read this submission and believe that I have an appropriate level of expertise to confirm that it is of an acceptable scientific standard.

# Reviewer report: Reviewer response for version 1

**DOI:** 10.5256/f1000research.176217.r369357

**Published:** 2025-03-24

**Authors:** Moin A Ansari

**Affiliations:** 1 Jazan University, Jazan, Jazan, Saudi Arabia

The authors have extended the concept of JU-algebras in fuzzy set theory to handle uncertainty in algebraic structures. JU-algebra is a class of logical algebras. Authors have studied Q-fuzzy sets to JU-subalgebras and JU-ideals in JU-algebra. 

 The paper is well-written and the results are mathematically true. This work can be considered as a contribution to the literature of logical algebras. However, the following revision is required.

 1. The study is based on JU-algebras. A separate paragraph for past and recent studies that have been done on JU-algebras must be provided in the introduction part possibly the second paragraph. This paragraph should include works based on JU-algebras specifically recent works on it. 

 2. Provide an example for each definition starting from Definition 1 to Definition 8 to make the study interesting for readers.

 3. In the conclusion part, write the possible directions for further studies that can be done based on your current work.

Is the work clearly and accurately presented and does it cite the current literature?

Yes

If applicable, is the statistical analysis and its interpretation appropriate?

Yes

Are all the source data underlying the results available to ensure full reproducibility?

Yes

Is the study design appropriate and is the work technically sound?

Yes

Are the conclusions drawn adequately supported by the results?

Yes

Are sufficient details of methods and analysis provided to allow replication by others?

Yes

Reviewer Expertise:

Algebras including logical and classical with its applications in different fields of science and technology

I confirm that I have read this submission and believe that I have an appropriate level of expertise to confirm that it is of an acceptable scientific standard, however I have significant reservations, as outlined above.

# Reviewer report: Reviewer response for version 1

**DOI:** 10.5256/f1000research.176217.r361133

**Published:** 2025-02-05

**Authors:** İbrahim Şanlıbaba

**Affiliations:** 1 Nevşehir Hacı Bektaş Veli University, Nevşehir, Turkey

Review Report on the paper:

***"*** Q-fuzzy structure on JU-algebra ***"***

 I gave the manuscript a thorough reading. The paper presents and specifies new kinds of fuzzy structures, in JU - algebras, to improve on classical JU-algebra theory, such as Q-fuzzy JU - subalgebras and Q-fuzzy JU-ideals. In order to open the door for more research into fuzzy algebra in mathematical and applied contexts, it also looks at important characteristics of these structures, such as their lower and upper level subsets, and looks at particular cases like Doubt and Normal Q-fuzzy structures.

 The outcomes are positive and satisfying, and the study appears to follow accepted scientific practices. The argument in the paper was constructed on a solid foundation of theory, concept, or other ideas. Additionally, the study or intellectual effort equivalent on which the publication is based was well planned. The technique is correctly applied. The article's substance is accurately and succinctly described in the abstract, and the conclusions and interpretations are supported by the data. But I wish to draw attention to these problems in the manuscript:

  

1. The motivation and originality of the article should be mentioned in the introduction section.
2. The novelty of the paper should be better emphasized in the introduction section.
3. Authors should better explain the advantages of the present method over the other numerical methods.
4. Conclusion part of the manuscript can be developed and more explanation about the results of the examples can be added. (Maybe the advantages of the present methods could be given in this section).
5. Please check entire manuscript carefully for grammatical errors and typos.
6. The importance of the design carried out in this manuscript can be explained better than other important studies published in this field. I recommend the authors to review other recently developed works. Şanlıbaba İ, et al., 2024 (Ref 1) and Şanlıbaba İ., 2025 (Ref 2), Şanlıbaba İ., 2022 (Ref 3)
7. Finally, please check the entire manuscript carefully about the above mentioned suggestions.

Last of all, this manuscript does have significant numerical methods content. The topic content of this paper is consistent with the goals of your journal. So my recommendation is **acceptance** of the manuscript after the **major** changes. It will hopefully add value to the existing literature.

Is the work clearly and accurately presented and does it cite the current literature?

Yes

If applicable, is the statistical analysis and its interpretation appropriate?

Yes

Are all the source data underlying the results available to ensure full reproducibility?

Yes

Is the study design appropriate and is the work technically sound?

Yes

Are the conclusions drawn adequately supported by the results?

Yes

Are sufficient details of methods and analysis provided to allow replication by others?

Yes

Reviewer Expertise:

Fuzzy set, fuzzy logic, Decision makin support, Artificial intelligient, Fuzzy soft set.

I confirm that I have read this submission and believe that I have an appropriate level of expertise to confirm that it is of an acceptable scientific standard, however I have significant reservations, as outlined above.

## References

[1] : A novel method for decision-making approach using multi-fuzzy soft systems with applications in analyzing the ımpact of two distinct drug categories. *Computational and Applied Mathematics* .2024;43(5) : 10.1007/s40314-024-02791-7 10.1007/s40314-024-02791-7

[2] : Uncredibility with fuzzy variables in uncertainty and its use in decision support in making sense of heart sounds. *Applied Soft Computing* .2025;169: 10.1016/j.asoc.2024.112641 10.1016/j.asoc.2024.112641

[3] : Similarity measurement of fuzzy entropies of respiratory sounds and risk measurement according to credibility distributions. *Soft Computing* .2022;26(19) : 10.1007/s00500-022-07415-y 10007-10017 10.1007/s00500-022-07415-y

# Response

**Published:** 2025-02-05

**Authors:** Selamawit Gelaw

**Affiliations:** Bahir Dar University, Ethiopia

I sincerely appreciate and thank you for your insightful suggestions and constructive comments, which have been invaluable in improving the quality of our work. 

  Herewith below  my response to reviewer comments

**1.** **The motivation and originality of the article should be mentioned in the introduction section**

 In the introduction, the motivation and originality is presented under the last paragraph. To explain clearly, JU-algebras have not been thoroughly explored in the context of Q-fuzzy sets, specifically with respect to JU-subalgebras and JU-ideals.  This gap motivates us to conduct this study, where we introduce the novel concepts of Q-fuzzy JU-subalgebras and Q-fuzzy JU-ideals to extend the theoretical understanding and practical applications of JU-algebras.

**2.** **The novelty of the paper should be better emphasized in the introduction section.**

 The novelty of the paper is mentioned in the introduction, basically we extends Q-fuzzy sets to JU-algebras, offering new insights and algebraic properties that were previously unexplored. **NOTE:  JU- algebra is a crisp set.**

**3.** **Authors should better explain the advantages of the present method over the other numerical methods.**

 The advantages of the present method is explained in the introduction with example how Q-fuzzy JU-subalgebras and Q-fuzzy JU-ideals allow for a more nuanced representation of fuzzy relationships, making them particularly well-suited for applications in fields like medicine, where conditions often do not have clear-cut boundaries. Furthermore, the inclusion of Doubt fuzzy sets enhances the robustness of the method, allowing for better handling of uncertain data.

**4.** **Conclusion part of the manuscript can be developed and more explanation about the results of the examples can be added. (Maybe the advantages of the present methods could be given in this section).**

 Yes we can explain more and our examples demonstrate how the method can be applied to real-world scenarios, but our focus is to highlight the theoretical concepts of  Q-fuzzy JU- sub algebra and Q- fuzzy JU- ideals of JU-algebra . Since we present examples for the theoretical illustration purpose only  so that adding results under conclusion is not as such important 

**6.** **The importance of the design carried out in this manuscript can be explained better than other important studies published in this field. I recommend the authors to review other recently developed works. Şanlıbaba İ, et al., 2024 (Ref 1) and Şanlıbaba İ., 2025 (Ref 2), Şanlıbaba İ., 2022 (Ref 3)"**

  I appreciate your nice work and the material you shared.  Yes this novel concepts of Q-fuzzy JU-sub algebras and Q-fuzzy JU-ideals demonstrates not only extends the theoretical frameworks of JU-algebras but also insights and invites other researchers work on practical applications in area like medical diagnostics, where precise and adaptable fuzzy models are critical for robust decision-making. However, at this time bringing and introducing this new theoretical concepts should come first.
